# Augmented Reality in Ophthalmology: Applications and Challenges

**DOI:** 10.3389/fmed.2021.733241

**Published:** 2021-12-10

**Authors:** Tongkeng Li, Chenghao Li, Xiayin Zhang, Wenting Liang, Yongxin Chen, Yunpeng Ye, Haotian Lin

**Affiliations:** ^1^State Key Laboratory of Ophthalmology, Zhongshan Ophthalmic Center, Sun Yat-sen University, Guangzhou, China; ^2^Zhongshan School of Medicine, Sun Yat-sen University, Guangzhou, China; ^3^Guangdong Eye Institute, Department of Ophthalmology, Guangdong Provincial People's Hospital, Guangdong Academy of Medical Sciences, Guangzhou, China; ^4^School of Biomedical Engineering, Sun Yat-sen University, Guangzhou, China; ^5^Center for Precision Medicine, Sun Yat-sen University, Guangzhou, China

**Keywords:** augmented reality, ophthalmology, therapy, education, clinical assistance

## Abstract

Augmented reality (AR) has been developed rapidly and implemented in many fields such as medicine, maintenance, and cultural heritage. Unlike other specialties, ophthalmology connects closely with AR since most AR systems are based on vision systems. Here we summarize the applications and challenges of AR in ophthalmology and provide insights for further research. Firstly, we illustrate the structure of the standard AR system and present essential hardware. Secondly, we systematically introduce applications of AR in ophthalmology, including therapy, education, and clinical assistance. To conclude, there is still a large room for development, which needs researchers to pay more effort. Applications in diagnosis and protection might be worth exploring. Although the obstacles of hardware restrict the development of AR in ophthalmology at present, the AR will realize its potential and play an important role in ophthalmology in the future with the rapidly developing technology and more in-depth research.

## Introduction

Augmented reality (AR) is a technology that enhances the natural environment with computer-generated information in real-time (1). AR is not restricted to the visual sense. It can be implemented for all feelings, including sight, hearing, smelling, and touching (2). Besides adding virtual information to the natural environment, AR applications include removing or processing real objects from the real environment, more commonly called mediated reality or diminished reality (1, 2). Unlike virtual reality (VR), which completely immerses users in a computer-generated virtual world, AR is based on the natural world and enhances the real environment with computer-generated information (1).

As a developing technology, AR has drawn the interest of researchers from different fields. Besides, AR has attracted companies like Google and Microsoft, which created AR devices such as Google Glass and HoloLens, providing hardware foundations for subsequent research. With the help of researchers and companies, AR has been developed rapidly and implemented in many fields such as medicine, maintenance, and cultural heritage (3).

Healthcare has become one of the pioneers, especially for applications requiring guidance and assistance (4). For example, AR has been certified effective in medical education and training, surgery navigation, and gastrointestinal endoscopy (5–7). However, unlike other specialties, ophthalmology connects closely with AR since most AR systems are based on vision generated by the eyes. Therefore, AR has particular applications in ophthalmology. Especially in the therapy of ocular diseases, AR possesses enormous potential to provide alternative or adjuvant choices in non-invasive and convenient ways to benefit patients who could not receive traditional medicine and surgical treatment. In addition to therapy, AR also has been implemented in education and clinical assistance in ophthalmology.

In this article, we reviewed AR in ophthalmology, summarized the applications and challenges of AR, and provided some suggestions for further research. We illustrated the structure of an AR system in ophthalmology, presented essential hardware, and systematically introduced AR's applications in ophthalmology, including applications in therapy, education, and clinical assistance. The ocular diseases that have been applied with AR in therapy include visual field defects, color vision deficiency, low vision, blindness, nyctalopia, metamorphopsia, and amblyopia. Applications in education contain medical education and public education. Applications in clinical assistance involve combining optical coherence tomography (OCT) and AR in surgery, deep anterior lamellar keratoplasty surgery navigation, and slit-lamps examination assistance.

## Structure of an AR System in Ophthalmology

### Overview

**Figure 1:**
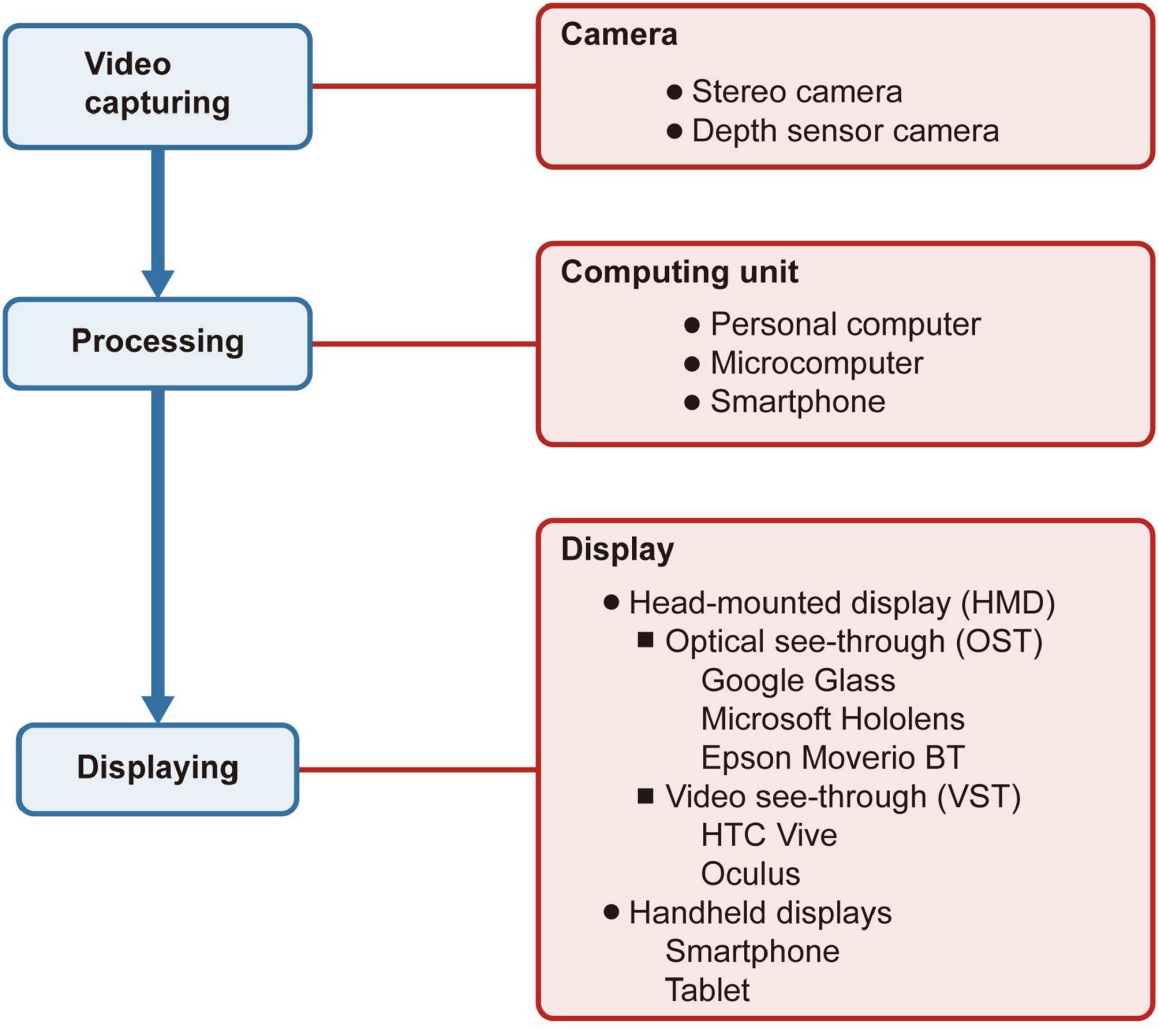
Structure of an augmented reality system in ophthalmology. The boxes filled in blue indicate the system's procedures, while the boxes in red indicate the devices connected with the procedures. The camera captures the natural environment and then transmits it to the computing unit for processing. Finally, the processed information is reflected on the display.

As shown in Figure 1, an AR system includes three primary modules: video capturing, processing, and displaying. The camera captures the natural environment and then transmits it to the computing unit for processing. Finally, the processed information is reflected on the display.

### Camera

There are many kinds of cameras for video capturing, depending significantly on the type of application. For instance, if developers need the function to evaluate the depth of the space, they probably will use a depth sensor camera. The stereo camera can support the 3D reconstruction of the real world.

### Computing Unit

The AR systems used to use personal computers as their computing center. With the invention of smartphones and microcomputers, the computing center became portable and subtle or directly embedded in the display.

### Display

The displays applied to AR in ophthalmology include head-mounted displays (HMDs) and handheld displays. HMDs are displays worn on the head to place images over the user's view. The display technologies can be video see-through (VST) or optical see-through (OST) (8). VST display uses video, which integrates virtual and natural environments to cover a complete view of the user, and the user cannot see the natural environment directly. On the contrary, the OST display only overlays the virtual images on the field of vision, and the user can see the natural environment as usual. The handheld display, like a smartphone, is a VST display with a small computing center held in the user's hands.

### Major AR Prototypes

There are two major AR prototypes: HMD-based AR systems and smartphone-based AR systems (9). HMD-based AR systems include AR systems produced by commercial companies like Google and Microsoft or homemade AR systems. As for some mainstream AR systems on the market, Google Glass, Microsoft HoloLens, and Epson Moverio BT series use OST display while the HTC Vive and Oculus use VST display. All of them have systems and give the users a mature experience. With the progress and maturity of technology, smartphones gradually grew into multipurpose tools, even replacing personal computers. With the miniature computing units and a high-resolution camera, smartphones can also be a platform to carry and achieve some AR applications. Compared with HMD-based AR systems, smartphone-based AR systems are more portable and cheaper, making it more widespread to promote smartphone-based AR systems (8). However, due to smartphones are handheld displays, they are not expected to be used for a long time. Using HMD is not only more comfortable than using a smartphone but also a better visual experience.

## Applications of AR in Ophthalmology

### Applications in Therapy

To meet the authoritative standard, we classified applications in therapy of ocular diseases according to the International Classification of Diseases 10th published by the World Health Organization^1^ The ocular diseases that have been applied with AR include visual field defects, color vision deficiency, low vision, blindness, nyctalopia, metamorphopsia, and amblyopia.

#### Visual Field Defects

Several ophthalmic diseases could cause visual field defects (VFD), including glaucoma, stroke, and retinitis pigmentosa (10). Visual field defects would bring difficulties in patients' daily life such as driving, crossing the road, reading, and visual searching (11–13). Due to the restricted vision field, the patients with VFD are less sensitive to surrounding dangers, threatening their health severely. In addition, some VFD are caused by brain injury, which cannot be reversed by traditional medicine or surgical treatment (14). Fortunately, the appearance of AR gives a considerable solution that provides a visual aid and improves searching capability.

**Figure 2:**
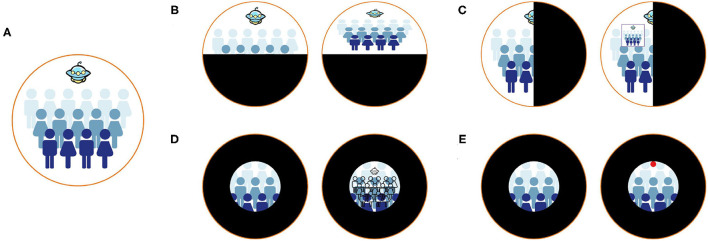
Different methods to aid patients with visual field defects. **(A)** Healthy vision. **(B)** The remapping method (10). Patients with visual field defects cannot see the entire scene. After remapping, patients can see the entire scene in their residual visual field. **(C)** The method of overlaying the overview window (15). After overlaying an overview window, patients can perceive the entire scene and natural vision simultaneously. **(D)** The method of using visual multiplexing (16). Patients can perceive the counter view of the entire scene and natural vision simultaneously. **(E)** The method of implementing danger indicators (17). Patients with tunnel vision cannot notice the danger the Unidentified Flying Object brought. The danger indicators can help patients notice surrounding dangers.

Image remapping, overlaid window, visual multiplexing, and danger indicator has been developed to provide a visual aid (Figure 2). Sayed et al. proposed customized digital spectacles with the image remapping method (10). The users were asked to measure their visual field at first. Then the images captured by the camera were remapped using resizing and shifting algorithms to adapt to the measured visual field (Figure 2B). A series of prospective case studies have been held to verify the functional field's efficiency (10, 18). However, digital spectacles cannot preserve the user's original vision. Another method implemented an overlaid window to display the overview scene captured by the camera (Figure 2C). The window is overlaid on the actual visual field of the user (15). Unfortunately, this method had an inherent contradiction between the augmented contextual information and local unscreened information since the overlaid window would inevitably block the natural view. Peli proposed visual multiplexing, defined as two or more signals transmitted on the same channel. In this way, the complete information can be used at the receiving end (19, 20). Spatial multiplexing, one of the visual multiplexing methods, has been implemented to aid tunnel vision (16). The minified edge images of the environmental scene were overlaid onto the user's natural vision (Figure 2D). The edge pixels did not block the realistic view since they only occupied a tiny part of a field of view. Another AR system is developed to notice patients with the tunnel vision of surrounding danger (17). The system would track the moving objects in real-time and extract their characteristics to determine their dangerous degree. The circles are superimposed on the edge of the visual field, and the perilous degree determines their color while moving objects assess their position (Figure 2E). Similarly, Ichinose et al. used edge indicator substitute circles to supplement the information in the lost vision of the patients (21).

Despite providing a visual aid for the VFD patient, improving searching capability is another direction. In Zhao's study, CueSee offered five visual cues for users who cannot finish visual search tasks (13). Among them, guidelines and sun rays are the cues designed for users with peripheral vision loss. The former connected the center of the display and the object with a red guideline, and the latter converged the center of the target with eight red guidelines. The location of the object could be indicated in these ways.

#### Color Vision Deficiency

Color vision deficiency (CVD), also known as color blindness, is a group of ophthalmic diseases that affect 8% of males globally. Patients have difficulties perceiving and distinguishing specific colors (22, 23). CVD brings obstacles to patients' daily life and restricts their occupations (24). Although CVD cannot be cured by medical treatment right now, the AR system could help users improve their ability to distinguishing colors and even perform close to healthy people (24).

Several commercial AR devices provide prototypes for CVD aiding research (24). Omnicolor and Chroma are applications based on Google Glass, one of the most popular intelligent glasses (25, 26). Popleteev's applications and Chroma glasses are based on Epson's Moverio, another AR device cheaper than Google Glass (27, 28). Besides, Schmitt et al. developed applications on the smartphone to help patients with CVD (29). Other researchers assemble homemade AR systems or modify existing devices (25, 26, 30).

**Figure 3:**
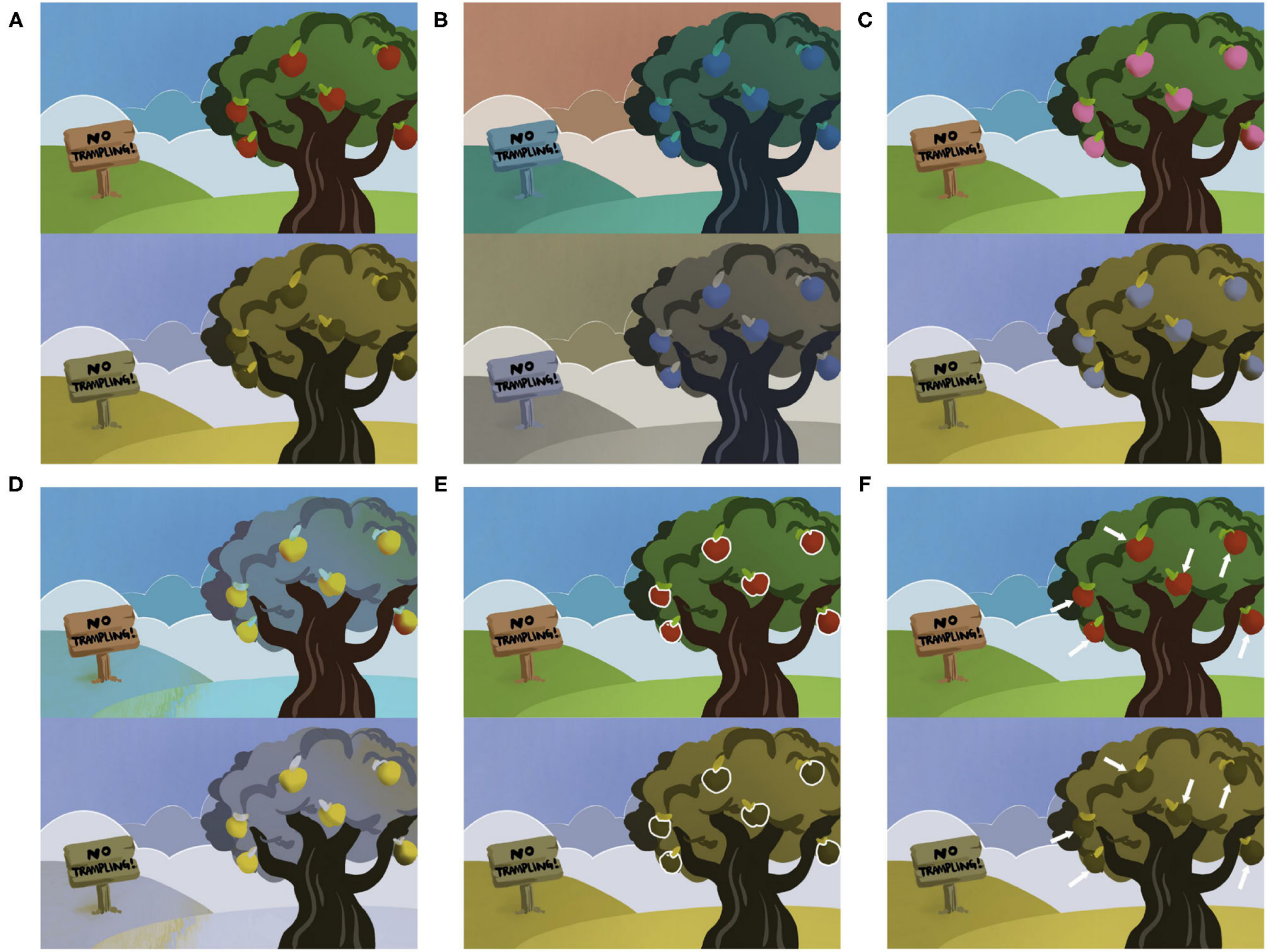
Different methods to help patients with color blindness. The top figure indicates healthy vision in each pair of figures, while the bottom indicates patients with protanopia. **(A)** The natural scene. The patients with protanopia cannot distinguish the apples in red and the leaves in green. **(B)** The daltonization method (27). All colors are shifted to achieve less confusing color combinations. **(C)** The highlight method (26). Once the patients want to distinguish any color, the target color will be replaced by other colors that are easier to distinguish. In this case, red is replaced by pink. **(D)** The contrast method (26). In this case, red is replaced by yellow, and green is replaced by blue. **(E)** The outline method (26). In this case, the areas in red are outlined in order to be distinguished easily. **(F)** The method of using icons (30). In this case, the areas in red are indicated by the arrows.

The processing technologies can be classified into two categories: substituting colors and augmenting visual information (Figure 3). Substituting colors is a group of strategies to transfer the target color to another one, including daltonization, highlight, and contrast. The most representative and popular algorithm is daltonization (Figure 3B), which attempts to shift colors to achieve less confusing color combinations for patients with CVD (25–27, 31, 32). Besides, the highlight method refers to highlight the target colors by replacing them with colors that are easier to see [(26, 28, 29); Figure 3C]. In addition, color contrast is a special algorithm for distinguishing a pair of colors (26, 28, 29). The mechanism of the algorithm is to transfer the target pair of colors to another particular pair of colors that are easier to distinguish (Figure 3D). Augmented visual information includes outline the shape of the area of target colors and indicates the target color with texts or icons [(26, 28–30); Figures 3E,F].

#### Blindness

**Figure 4:**
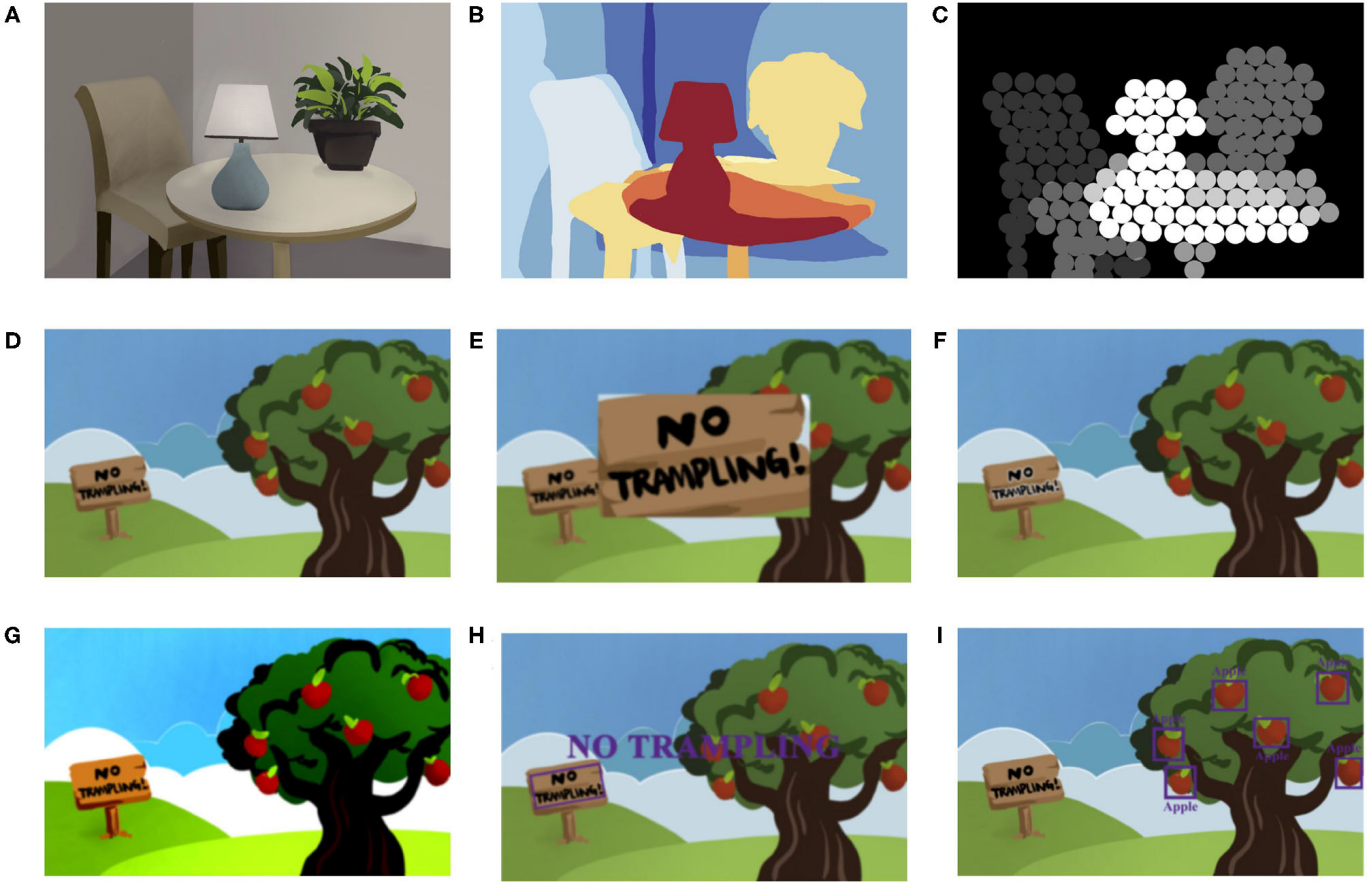
Aiding the patients with low vision and blindness. **(A–C)** Illustrate using distance-based vision aid AR system to help the blind. **(A)** The natural scene. **(B)** The method using colors to indicate distances (37). The area in warmer colors indicates the closer distances, while the area in cooler colors indicates the farther. **(C)** The method using brightness to indicate distances (36). Brighter indicates closer distance while darker indicates farther. **(D–I)** Illustrate different methods to aid the patients with low vision (39). **(D)** The natural vision of patients with low vision. **(E)** The magnification method. **(F)** The edge enhancement method. **(G)** The contrast enhancement method. **(H)** The text extraction method. **(I)** The object recognition method.

Blindness is defined as the best-corrected visual acuity of a patient's better-seeing eye is <20/400 (33). The dominant causes of blindness are age-related diseases, such as age-related macular degeneration (34). Up to now, there is no therapy that can reverse blindness since the diseases disturb the transmission of visual data from the eye to the brain. Blindness brings severe obstacles to the patient's life, especially restricting their mobility. Perceiving the surrounding environment and avoiding obstacles are essential for improving mobility. Therefore, several distance-based vision aid AR systems have been proposed (35–38). These AR systems make use of color perception, light perception, and hearing to convey information. For patients with blindness who still can perceive color vision, two studies use colors to indicate distance (Figures 4A,B). Their system is applied on Microsoft HoloLens, calculating the distances between objects and users based on the video stream (35, 37). Similarly, for patients considered blind but retain light perception, Hick et al. developed an HMD using the brightness of the light-emitting diodes to inform the patients about the distance (36). While the distance between object and user was shortened, the light-emitting diodes would be bright (Figure 4C). For total blindness, the spatialized sound was used to express the distances in Liu's study (38). The intensity of the sound increased as the distance shortened.

#### Low Vision

Low vision is defined as the best-corrected visual acuity of a patient's better-seeing eye is better than 20/400 and <20/60 (33). Patients with low vision have difficulties in recognizing things such as reading. The applications of AR in low vision aid focus on strengthening the recognizing capability in different strategies, including magnification, edge enhancement, contrast enhancement, object recognition, and text extraction. Magnification is the single most common strategy (39–43). The images after magnification are showed in a window or covered the user's sight (Figures 4D,E). However, both of them are inevitable to reduce the field of view. Therefore, some research adjusts the transparency of the magnified images for the patients to see the real environment and magnified images together (40, 43). Besides, edge enhancement can avoid this trouble, enhancing the edge of the objects while remaining the view of the users [(13, 39, 42, 44); Figure 4F]. Hwang and Peli used the positive and negative Laplacian filters to enhance the edges (44). The former one would highlight the edge with clear surroundings, while the latter one is the opposite. In their AR system, the users can choose one of three levels to enhance the edge according to their situation. In addition, contrast enhancement helps users recognize things by increasing the contrast of the images [(13, 39, 42); Figure 4G]. In Zhao's study, the contrast enhancement methods include maintaining the hues while increasing the contrast and image binarization processing (39). In recent years, with the rapid development of artificial intelligence, especially convolutional neural networks, breakthroughs have been made in image recognition (45). Among them, object or facial recognition technology [(39, 42, 45); Figure 4H] and text extraction [(39, 40, 46); Figure 4I] using optical character recognition have been combined with AR as strategies to improve recognizing capability of the user with low vision. After object or facial recognition and text extraction, the AR systems return with audio feedback and text information.

#### Nyctalopia, Metamorphopsia, and Amblyopia

**Figure 5:**
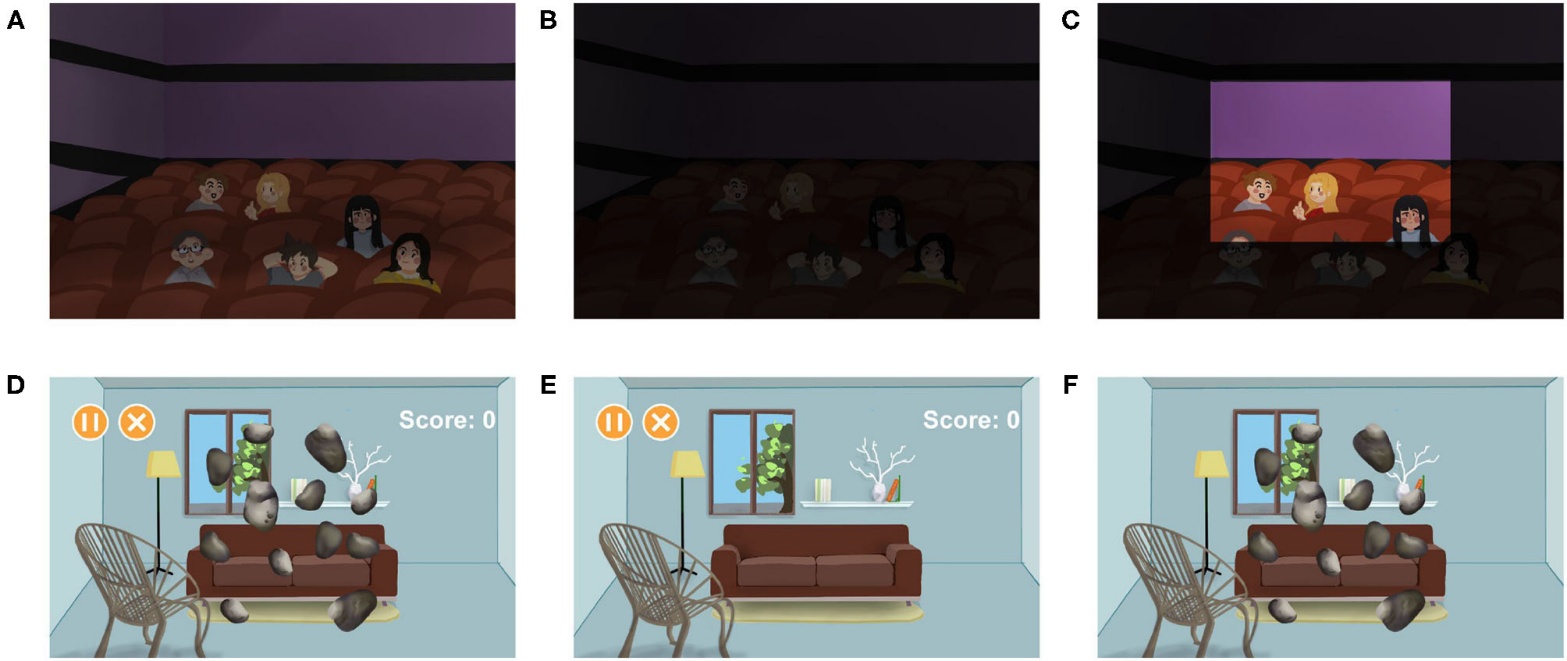
Aiding patients with amblyopia and nyctalopia. **(A–C)** Illustrate aiding patients with nyctalopia (48). **(A)** The healthy vision. **(B)** The vision of patients with nyctalopia. **(C)** The vision of patients with nyctalopia after using the AR systems. The part of the scene is brightened to help the patients. **(D–F)** Illustrate aiding patients with amblyopia (49). The patients are forced to use their lazy eyes more important than healthy eyes. In this case, patients play games that ask them to crush the roaming stones. **(D)** The binocular vision. **(E)** The vision of the healthy eye, containing the game menus that do not need too much attention. **(F)** The vision of the lazy eye, containing roaming stones that require high concentration.

In contrast to color blindness, nyctalopia has problems with rods rather than cones (47). Therefore, patients with nyctalopia cannot recognize things clearly in a dark environment (Figures 5A,B). AR has offered a new way in the therapy of nyctalopia by brightening the vision in real-time. Hu et al. proposed a night vision enhancement based on see-through glasses (48). The glasses first inverted the dark image, then used the de-hazing algorithms to process the inverted image. After that, the processed image will be resized and calibrated to the real environment (Figure 5C). Another research developed Troyoculus, which used the self-illumination filter to brighten the video streaming and implemented a bright excess filter to prevent bright excess (50). Troyoculus was developed on two prototypes, HMD and smartphone.

Metamorphopsia is a group of macular disorders that cause the patient's sight to twist (51). The common causes of metamorphopsia include age-related macular degeneration, diabetic macular edema, and vitreoretinal interface disorders. Bozzelli developed an AR system that compensated or reduced visual geometric distortions caused by metamorphopsia in real-time, according to the precise measurement (52). The system tested and adjusted its algorithms constantly until the user's vision was corrected. The correction algorithm mapped the video streams onto a polygonal mesh and offset its vertices.

Amblyopia is a disorder that affects the spatial vision of monocular or binocular (53). It is a developmental disease caused by strabismus or anisometropia. Several VR and AR systems are developed to correct amblyopia by providing games that force users to use their lazy eyes more important than healthy eyes (Figures 5D–F). While using the VR systems, users are completely immersed in the virtual environment, which increases the occurrence of accidents. On the contrary, users can see the real environment while using the AR system. Therefore, AR is considered as a better substitution than VR in amblyopia therapy (49).

### Applications in Education

#### Medical Education

Ophthalmic surgery and diagnosis are difficult to get started due to their meticulousness. As a result, medical students need to practice a lot to be qualified. However, many students cannot get enough chances to practice because of the lack of cases. The surgical or diagnostic training simulators based on VR or AR systems have been presented to solve this problem. Students can acquire adequate practice through simulators to improve surgical or diagnostic quality. An inevitable defect of the VR simulators is that VR blocks the connection with the real environment. On the contrary, the AR simulators can reserve the real environment while simulating the surgical or diagnostic information. The AR simulators have been implemented in direct or indirect ophthalmoscopy and microsurgery. Schuppe et al. presented EYESI, a training simulator for indirect ophthalmoscopy, in 2009 for the first time (54). EYESI could show the hands of the examiner from the real world while simulating the patient and fundus. Besides, the AR training simulator of indirect ophthalmoscopy has been developed on low-cost mobile platforms (55). The simulator has two prototypes. One is based on the smartphone used as a direct ophthalmoscope, and the other is based on HMD. Although it was helpful for users to learn ophthalmoscopy, most participants thought they could not master this technology. Moreover, Ropelato et al. developed an AR system for training micromanipulation skills, equipped with a simulative training environment and assessing system (56). In this system, Microsoft HoloLens presented a surgical environment that allowed users to stay in touch with the real world, such as the real instruments or the assistants involved in the surgical procedure. Similarly, CatAR was developed for cataract surgical training (57). What had improved was that they updated the display's resolution, which improved the reality of the simulated surgery.

#### Public Education

Since ophthalmic diseases might cause severe consequences and significantly impact life quality, prevention is more important than treatment (58). Besides, although the public might know a little about ophthalmology, a healthy person might not understand how badly the patients suffer from ocular diseases (59). As a result, it is necessary to educate and inform the public. The following research has developed several devices to simulate ophthalmic diseases. Ates et al. presented a low-cost simulation named SIMVIZ (60). Two wide-angle cameras catch the real world, and then the filters deal with the video stream in different ways according to different disease modes and finally send it to the user's sight. The filters can simulate macular degeneration, diabetic retinopathy, glaucoma, cataracts, color blindness, and diplopia using different algorithms. However, SIMVIZ provides an immersive simulative environment for users. It has problems of inconvenience, low resolution, and accuracy. Similarly, Jones et al. proposed methods to simulate six vision, including disability glare, blur, metamorphopsia, perceptual filling-in, and color vision deficits (61). These methods are implemented in smartphones and HMD. In subsequent research, Jones proved the effectiveness of these methods (62). Moreover, an eye-tracked AR system was developed to improve the accuracy of simulated cataracts (63). The processing system included the following parts: reducing visual acuity, reducing contrast, applying color shift, simulating dark shadows, and simulating sensitivity to light. In order to find the best parameters, they conducted interactive experiments with cataract patients. The cataract patients had undergone surgery on one eye while the other had not. They were asked to compare the simulation to their cataract view and return the result to adjust the parameters. In the end, several parameters were constantly adjusted to attach the best simulation. Furthermore, simulating the vision of patients with ocular diseases can enlarge the sample size for research (64).

### Applications in Clinical Assistance

Surgery and diagnosis are important but challenging in ophthalmology since they require a quantity of experience (65). Except for helping medical students practice in the classroom, AR has been implemented in real-time clinical assisting. The AR system using additional information such as imaging information or diagnostic standard to improve the quality of surgery or diagnosis. OCT is a technique that can obtain high tissue resolution images, and the optical microscope and OCT can share the same optical path (66). As a result, it has great potential to be introduced to ophthalmic surgery with AR systems. Combining AR and OCT in ophthalmic surgery has been explored to improve the accuracy of the surgery (67, 68). An advantage of OCT is that it can perform three-dimensional reconstruction of scanned images and provide any section image or depth stereo image at surgeons' will. Through the AR system, the OCT images can be integrated with the surgery scene in real-time, and the surgeon can investigate the OCT images and surgery scene simultaneously. In Roodaki et al.'s study, the vertical section was provided to inform the surgeon of the distance between surgical instruments and fundamental ocular tissues (67). In another study, different depths of the stereo image were used to provide vivid information on tissues (68). Besides, the AR system has been implemented in deep anterior lamellar keratoplasty surgery navigation. The system carrying artificial intelligence can detect the corneal counter and overlay it onto the video streaming, assisting the surgeons in recognizing the corneal. Furthermore, AR has been introduced in slit-lamp for assisting diagnosis (69). The images stored previously were placed onto the real-time slit-lamp right-view while the left-view of the slit-lamp remains natural. The users can improve their diagnostic accuracy by comparing the natural view with the standard.

## Discussion

AR is a popular technology in ophthalmology and has developed rapidly in the recent decade since the appearance of commercial HMDs and smartphones. The applications of AR in ophthalmology introduced in this review involve therapy, education, and clinical assistance. In order to have a better understanding of AR's development in ophthalmology, we have counted the number of publications in each application according to our classification method in the Google Scholar database (Supplementary Figure 1 and Supplementary Table 1). The AR's applications in ophthalmic therapy account for the largest share, followed by education and clinical assistance. Although the number of studies on therapy is the largest, the research of AR in ophthalmic therapy is unbalanced. There are 14 publications about low vision, but only 1–2 studies on nyctalopia, metamorphopsia, and amblyopia. It requires researchers to pay more attention to AR's applications in ocular diseases lacking research. In education, although the amount of publications in medical education is limited, the AR systems are well-evaluated. For example, EYESI, a surgical simulator. A systematic review reported that 38 publications had held evaluation experiments on it (70). As a result, the development of AR in medical education is more mature than in other fields in ophthalmology. In addition, few researchers study clinical assistance, especially diagnostic assistance. Although it is of great significance to promote the progress of the overall medical level in ophthalmology, a lot of research is needed.

Applications in therapy aim to improve patients' vision or activities closer to healthy people. The common symptoms of eye disease are visual disturbances, and AR equips the ability to process visual information to enhance vision. Therefore, the therapy is a unique application in ophthalmology compared to other diseases. Besides, as a non-invasive treatment method, AR has great potential in alternative therapy. For patients with ocular diseases who cannot tolerate surgery or drugs, AR is a better choice. In addition, AR provides visual aids to patients suffering from incurable diseases to improve their quality of life. However, efforts are still needed to pay in ophthalmology treatment. Most AR applications in ophthalmology still lack adequate clinical research to evaluate the effectiveness, especially in ophthalmic disease aid. Although a few of research have held evaluation, the problems with insufficient sample size and selective bias still exist (42, 62). It is urgent to hold clinical experiments on evaluating AR applications in ophthalmology to provide robust evidence to accelerate their widespread. Besides, a highly customized AR system implemented in ophthalmic disease aid is expected to develop. At present, AR systems implemented in ophthalmic disease aid mainly focus on a specific disease. However, a patient may suffer from multiple diseases at the same time. For example, a patient suffering from myopia and color blindness needs to wear prescription glasses while using the AR system (25). As a result, the combination of different AR applications in ophthalmic diseases should be considered. In addition, the current applications of AR in fundus diseases such as age-related macular degeneration cannot solve the problem from the root cause because the visual information provided by AR is disturbed or hindered in the transmission of the retina or visual pathway. If it is possible to transmit visual information bypassing the retina or even bypassing the entire visual pathway to achieve the cerebral cortex, patients might recover to healthy levels. However, it requires landmark breakthroughs in Neurobionics and the brain-computer interface (71). For now, AR can be considered exploring the therapy of ocular diseases by changing visual habits. The implementation of AR in amblyopia therapy provides a good example (49). Similarly, AR equips excellent potential for intervention to form healthy vision for ocular diseases related to visual development and formation.

The applications of AR in ophthalmic education involve public education and medical education in this review. Their common mechanism is simulating the vision vividly in real-time. The applications of AR in education can provide medical students with a lot of opportunities for practice. AR also allows healthy people to experience the vision of patients. In addition, compared to VR, the vision of AR simulation is more realistic and vivid because it is based on the natural environment. However, the evaluation of AR simulators is still lacking (70). In public education, obstacles still exist in evaluating several disease simulations, such as simulating the vision of color blindness. Since the vision of color blind patients cannot be obtained, precise evaluation standards have been lacked (72).

Similarly, the applications of AR in clinical assistance lacks effective evaluation. At present, AR is providing additional information to guide clinical activities in order to improve accuracy. However, AR technology includes not only adding information but also reducing and processing information. Therefore, AR technology can also be considered to remove things overlaying surgery or inspection targets in vision.

In addition to the three areas summarized in this article, AR also has potential in diagnosing and protection. There have been some researches well-developed on VR but lack exploring in AR in the diagnosis of strabismus (73). It might be possible to achieve better effectiveness by implementing well-developed diagnosis applications from VR to AR since AR is based on natural vision and can be used for a long time (9). The development of a monitoring system based on AR devices can monitor eye health in real-time and discover hidden diseases. In addition to treating patients with eye diseases, the AR system can be considered to protect healthy people and patients suffering from surgery or treatment. For healthy people, the AR system can be used to process vision in situations that are harmful to the eyes. For instance, when driving under strong sunlight, the brightness of the corresponding field of view is expected to be reduced with an AR system. The AR system can be implemented to prevent possible secondary ocular diseases for patients suffering surgery or treatment. For example, patients who use atropine to dilate their pupils can use AR systems to avoid too bright vision.

Since AR applications in ophthalmology integrate medicine and engineering, the development of AR in engineering is also critical. The most significant restriction of the ophthalmic AR system is the hardware. The resolution of the image is an unavoidable question troubling cameras and displays. A 20/20 vision requires a display system with 60 pixels per degree resolution in theory (74). Most of the research is based on HMDs and smartphones, but these devices can only provide ~10–12 pixels per degree (57). Besides, the computing power and volume of the computing units restrict the function and mobility of the AR system. In the beginning, the AR system relies on a PC (36). As a result, it is bulky for users to undertake. With the rise of HMDs and smartphones, which use embedded computing centers, the mobility of AR systems has been significantly improved, but the small space limits its computing power. Fortunately, in the stage of Industry 4.0, the 5G telecommunication provides a massive capacity for real-time information transmission, which allows real-time cloud computing (75). The cloud computing method can reduce the volume immensely and increase the computing power (76). In addition, some HMDs are heavy, thus making them uncomfortable to use for a long time (77). Furthermore, the battery capacity is limited, restricting the using time (77). It is a hazard for ophthalmic patients that the AR system strikes in some time. This situation could be prevented by developing battery technology and multi-energy power (78).

In conclusion, applications of AR in ophthalmology have been implemented in therapy, education, and clinical assistance. However, there is still a large room for development, which needs researchers to pay more effort. Applications in diagnosis and protection might be worth exploring. Although the obstacles of hardware restrict the development of AR in ophthalmology at present, the AR will realize its potential and play an important role in ophthalmology in the future with the rapidly developing technology and more in-depth research.

## Author Contributions

HL, TL, and XZ designed the study. TL, CL, and XZ co-wrote the manuscript. WL, YC, and YY discussed and edited the paper. HL supervised this study. All authors discussed the results and commented on the paper.

## Funding

This study was funded by the National Natural Science Foundation of China (81770967 and 81822010). The funders had no role in the study design, interpretation, and writing of the paper.

## Conflict of Interest

The authors declare that the research was conducted in the absence of any commercial or financial relationships that could be construed as a potential conflict of interest.

## Publisher's Note

All claims expressed in this article are solely those of the authors and do not necessarily represent those of their affiliated organizations, or those of the publisher, the editors and the reviewers. Any product that may be evaluated in this article, or claim that may be made by its manufacturer, is not guaranteed or endorsed by the publisher.

## Footnotes

^1^*International Classification of Diseases*. Available online at: https://icd.who.int/browse10/2019/en#/H53-H54

## Abbreviations

AR: augmented reality
CVD: color vision deficiency
HMD: head-mounted displays
OCT: optical coherence tomography
OST: optical see-through
VFD: visual field defects
VST: video see-through.

## References

[1] Carmigniani J Furht B Anisetti M Ceravolo P Damiani E Ivkovic M. Augmented reality technologies, systems and applications. Multimed Tools Appl. (2011) 51:341–77. 10.1007/s11042-010-0660-6

[2] Azuma R Baillot Y Behringer R Feiner S Julier S MacIntyre B. Recent advances in augmented reality. IEEE Comput Graph Appl. (2001) 21:34–47. 10.1109/38.96345927295638

[3] Wang X Ong SK Nee AYC. A comprehensive survey of augmented reality assembly research. Adv Manufact. (2016) 4:1–22. 10.1007/s40436-015-0131-4

[4] Sielhorst T Feuerstein M Navab N. Advanced medical displays: a literature review of augmented reality. J Display Technol. (2008) 4:451–67. 10.1109/JDT.2008.200157527295638

[5] Lee K. Augmented reality in education and training. TechTrends. (2012) 56:13–21. 10.1007/s11528-012-0559-3

[6] Mahmud N Cohen J Tsourides K Berzin TM. Computer vision and augmented reality in gastrointestinal endoscopy. Gastroenterol Rep. (2015) 3:179–84. 10.1093/gastro/gov027 26133175 PMC4527270

[7] Vávra P Roman J Zonča P Ihnát P Němec M Kumar J. Recent development of augmented reality in surgery: a review. J Healthc Eng. (2017) 2017:4574172. 10.1155/2017/4574172 29065604 PMC5585624

[8] Van Krevelen D Poelman R. A survey of augmented reality technologies, applications and limitations. Int J Virt Real. (2010) 9:1–20. 10.20870/IJVR.2010.9.2.2767

[9] Manuri F Sanna A. A survey on applications of augmented reality. ACSIJ Adv Comput Sci Int J. (2016) 5:18–27. 10.1109/ICIIP47207.2019.898577927295638

[10] Sayed AM Kashem R Abdel-Mottaleb M Roongpoovapatr V Eleiwa TK Abdel-Mottaleb M. Toward improving the mobility of patients with peripheral visual field defects with novel digital spectacles. Am J Ophthalmol. (2020) 210:136–45. 10.1016/j.ajo.2019.10.005 31606442 PMC7002240

[11] Hirooka K Sato S Nitta E Tsujikawa A. The relationship between vision-related quality of life and visual function in glaucoma patients. J Glaucoma. (2016) 25:505–9. 10.1097/IJG.0000000000000372 26766401 PMC4888932

[12] Ong YH Jacquin-Courtois S Gorgoraptis N Bays PM Husain M Leff AP. Eye-search: a web-based therapy that improves visual search in hemianopia. Ann Clin Transl Neurol. (2015) 2:74–8. 10.1002/acn3.154 25642437 PMC4301677

[13] Zhao Y Szpiro S Knighten J Azenkot S. CueSee: exploring visual cues for people with low vision to facilitate a visual search task. In: 2016 ACM International Joint Conference on Pervasive and Ubiquitous Computing. Heidelberg (2016). p. 73–84. 10.1145/2971648.2971730

[14] Dhital A Pey T Stanford MR. Visual loss and falls: a review. Eye. (2010) 24:1437–46. 10.1038/eye.2010.60 20448666

[15] Zhao X Go K Kashiwagi K Toyoura M Mao X Fujishiro I. Computational alleviation of homonymous visual field defect with OST-HMD: the effect of size and position of overlaid overview window. In: 2019 International Conference on Cyberworlds (CW). Kyoto (2019). p. 175–82. 10.1109/CW.2019.00036

[16] Apfelbaum HL Apfelbaum DH Woods RL Peli E. Inattentional blindness and augmented-vision displays: effects of cartoon-like filtering and attended scene. Ophthal Physiol Opt. (2008) 28:204–17. 10.1111/j.1475-1313.2008.00537.x 18426419 PMC2396779

[17] Younis O Al-Nuaimy W Rowe F. A hazard detection and tracking system for people with peripheral vision loss using smart glasses and augmented reality. Int J Adv Comput Sci Appl. (2019) 10:1–9. 10.14569/IJACSA.2019.010020130959756

[18] Sayed AM Abdel-Mottaleb M Kashem R Roongpoovapatr V Elsawy A Abdel-Mottaleb M. Expansion of peripheral visual field with novel virtual reality digital spectacles. Am J Ophthalmol. (2020) 210:125–35. 10.1016/j.ajo.2019.10.006 31626763 PMC7002244

[19] Peli E. Vision multiplexing: an engineering approach to vision rehabilitation device development. Optom Vis Sci. (2001) 78:304–15. 10.1097/00006324-200105000-00014 11384008

[20] Peli E. Vision multiplexing: an optical engineering concept for low-vision aids. In: Proceedings SPIE 6667, Current Developments in Lens Design and Optical Engineering VIII. Vol. 66670C. San Diego, CA (2007). 10.1117/12.729315

[21] Ichinose K Fujishiro I Kashiwagi K Mao X Zhao X Toyoura M. Visual field loss compensation for homonymous hemianopia patients using edge indicator. In: 2020 International Conference on Cyberworlds (CW). Caen (2020). p. 79–85. 10.1109/CW49994.2020.00019

[22] El Moussawi Z Boueiri M Al-Haddad C. Gene therapy in color vision deficiency: a review. Int Ophthalmol. (2021) 41:1917–27. 10.1007/s10792-021-01717-0 33528822

[23] Keene DR. A review of color blindness for microscopists: guidelines and tools for accommodating and coping with color vision deficiency. Microsc Microanal. (2015) 21:279–89. 10.1017/S1431927615000173 25739321

[24] Stoianov M de Oliveira MS dos Santos Ribeiro MCL Ferreira MH de Oliveira Marques I Gualtieri M. The impacts of abnormal color vision on people's life: an integrative review. Qual Life Res. (2019) 28:855–62. 10.1007/s11136-018-2030-1 30443703

[25] Lausegger G Spitzer M Ebner M. OmniColor–a smart glasses app to support colorblind people. Int J Interact Mobile Technol. (2017) 11:161–77. 10.3991/ijim.v11i5.6922

[26] Tanuwidjaja E Huynh D Koa K Nguyen C Shao C Torbett P. Chroma: a wearable augmented-reality solution for color blindness. In: 2014 ACM International Joint Conference on Pervasive and Ubiquitous Computing. Seattle, WA (2014). p. 799–810. 10.1145/2632048.2632091

[27] Langlotz T Sutton J Zollmann S Itoh Y Regenbrecht H. Chromaglasses: computational glasses for compensating colour blindness. In: Paper Presented at the Proceedings of the 2018 CHI Conference on Human Factors in Computing Systems. Montreal, QC (2018). p. 1–12. 10.1145/3173574.3173964

[28] Popleteev A Louveton N McCall R. Colorizer: smart glasses aid for the colorblind. In: Proceedings of the 2015 Workshop on Wearable Systems and Applications. Florence (2015). p. 7–8. 10.1145/2753509.2753516

[29] Schmitt S Stein S Hampe F Paulus D. Mobile services supporting color vision deficiency. In: 2012 13th International Conference on Optimization of Electrical and Electronic Equipment (OPTIM). Brasov (2012). p. 1413–20. 10.1109/OPTIM.2012.6231860

[30] Dheeraj K Jilani SAK JaveedHussain MS. Real-time automated guidance system to detect and label color for color blind people using raspberry Pi. SSRG Int J Electron Commun Eng. (2015) 2:11–4. 10.14445/23488549/IJECE-V2I11P103

[31] Melillo P Riccio D Di Perna L Di Baja GS De Nino M Rossi S. Wearable improved vision system for color vision deficiency correction. IEEE J Transl Eng Health Med. (2017) 5:1–7. 10.1109/JTEHM.2017.2679746 28507827 PMC5418066

[32] Tang Y Zhu Z Toyoura M Go K Kashiwagi K Fujishiro I. Arriving light control for color vision deficiency compensation using optical see-through head-mounted display. In: 16th ACM SIGGRAPH International Conference on Virtual-Reality Continuum and its Applications in Industry. Tokyo (2018). p. 1–6. 10.1145/3284398.3284407

[33] World Health Organization. International Classification of Impairments, Disabilities, and Handicaps: A Manual of Classification Relating to the Consequences of Disease. Geneva: World Health Organization (1980). Available online at: https://apps.who.int/iris/bitstream/handle/10665/41003/9241541261_eng.pdf (accessed June 29, 2021).

[34] Scholl HP Strauss RW Singh MS Dalkara D Roska B Picaud S. Emerging therapies for inherited retinal degeneration. Sci Transl Med. (2016) 8:368rv6. 10.1126/scitranslmed.aaf2838 27928030

[35] Angelopoulos AN Ameri H Mitra D Humayun M. Enhanced depth navigation through augmented reality depth mapping in patients with low vision. Sci Rep. (2019) 9:11230. 10.1038/s41598-019-47397-w 31375713 PMC6677879

[36] Hicks SL Wilson I Muhammed L Worsfold J Downes SM Kennard C. A depth-based head-mounted visual display to aid navigation in partially sighted individuals. PLoS ONE. (2013) 8:e67695. 10.1371/journal.pone.0067695 23844067 PMC3701048

[37] Kinateder M Gualtieri J Dunn MJ Jarosz W Yang XD Cooper EA. Using an augmented reality device as a distance-based vision aid—promise and limitations. Optom Vis Sci. (2018) 95:727–37. 10.1097/OPX.0000000000001232 29877901 PMC6133229

[38] Liu Y Stiles NR Meister M. Augmented reality powers a cognitive assistant for the blind. Elife. (2018) 7:e37841. 10.7554/eLife.37841 30479270 PMC6257813

[39] Zhao Y Szpiro S Azenkot S. Foresee: a customizable head-mounted vision enhancement system for people with low vision. In: 17th International ACM SIGACCESS Conference on Computers and Accessibility. Lisbon (2015). p. 239–49. 10.1145/2700648.2809865

[40] Bakshi AM Simson J de Castro C Yu CC Dias A. Bright: an augmented reality assistive platform for visual impairment. In: The 2019 IEEE Games, Entertainment, Media Conference (GEM). New Haven, CT (2019). p. 1–4. 10.1109/GEM.2019.8811556

[41] Gonçalves P Orlosky J Machulla TK. An augmented reality assistant to support button selection for patients with age-related macular degeneration. In: 2020 IEEE Conference on Virtual Reality and 3D User Interfaces Abstracts and Workshops (VRW). Atlanta, GA (2020) p. 730–1. 10.1109/VRW50115.2020.00216

[42] Min Htike HH Margrain T Lai YK Eslambolchilar P. Augmented reality glasses as an orientation and mobility aid for people with low vision: a feasibility study of experiences and requirements. In: 2021 CHI Conference on Human Factors in Computing Systems. Yokohama (2021). p. 1–15. 10.1145/3411764.3445327

[43] Stearns L Findlater L Froehlich JE. Design of an augmented reality magnification aid for low vision users. In: 20th International ACM SIGACCESS Conference on Computers and Accessibility. Galway (2018). p. 28–39. 10.1145/3234695.3236361

[44] Hwang AD Peli E. An augmented-reality edge enhancement application for Google Glass. Optom Vis Sci. (2014) 91:1021. 10.1097/OPX.0000000000000326 24978871 PMC4111789

[45] Lang F Schmidt A Machulla T. Augmented reality for people with low vision: symbolic and alphanumeric representation of information. In: The International Conference on Computers Helping People with Special Needs. Lecco (2020). p. 146–56. 10.1007/978-3-030-58796-3_19

[46] Huang J Kinateder M Dunn MJ Jarosz W Yang XD Cooper EA. An augmented reality sign-reading assistant for users with reduced vision. PLoS ONE. (2019) 14:e0210630. 10.1371/journal.pone.0210630 30650159 PMC6334915

[47] Almutairi F Almeshari N Ahmad K Magliyah MS Schatz P. Congenital stationary night blindness: an update and review of the disease spectrum in Saudi Arabia. Acta Ophthalmol. (2020) 99:581–91. 10.1111/aos.14693 33369259

[48] Hu C Zhai G Li D. An augmented-reality night vision enhancement application for see-through glasses. In: 2015 IEEE International Conference on Multimedia and Expo Workshops (ICMEW). Turin (2015). p. 1–6. 10.1109/ICMEW.2015.7169860

[49] Nowak A Wozniak M Pieprzowski M Romanowski A. Towards amblyopia therapy using mixed reality technology. In: Paper presented at the 2018 Federated Conference on Computer Science and Information Systems (FedCSIS). Poznan (2018). p. 279–82. 10.15439/2018F335

[50] Fernandez A Fernandez P López G Calderón M Guerrero LA. Troyoculus: an augmented reality system to improve reading capabilities of night-blind people. In: The International Work-Conference on Ambient Assisted Living. Puerto Varas (2015). p. 16–28. 10.1007/978-3-319-26410-3_3

[51] Midena E Vujosevic S. Metamorphopsia: an overlooked visual symptom. Ophthalmic Res. (2016) 55:26–36. 10.1159/000441033 26554918

[52] Bozzelli G De Nino M Pero C Ricciardi S. AR based user adaptive compensation of metamorphopsia. In: Paper Presented at the Proceedings of the International Conference on Advanced Visual Interfaces. Salerno (2020). p. 1–5. 10.1145/3399715.3399929

[53] Maurer D Mc KS. Classification and diversity of amblyopia. Vis Neurosci. (2018) 35:E012. 10.1017/s0952523817000190 29905124

[54] Schuppe O Wagner C Koch F Manner R. EYESi ophthalmoscope–a simulator for indirect ophthalmoscopic examinations. Stud Health Technol Inform. (2009) 142:295–300. 10.3233/978-1-58603-964-6-295 19377172

[55] Acosta D Gu D Uribe-Quevedo A Kanev K Jenkin M Kapralos B. Mobile e-training tools for augmented reality eye fundus examination. In: Interactive Mobile Communication, Technologies and Learning. Hamilton, ON (2018). p. 83–92. 10.1007/978-3-030-11434-3_13

[56] Ropelato S Menozzi M Michel D Siegrist M. Augmented reality microsurgery: a tool for training micromanipulations in ophthalmic surgery using augmented reality. Simul Healthc. (2020) 15:122–7. 10.1097/SIH.0000000000000413 32044852

[57] Huang YH Chang HY Yang WL Chiu YK Yu TC Tsai PH. CatAR: a novel stereoscopic augmented reality cataract surgery training system with dexterous instruments tracking technology. In: 2018 CHI Conference on Human Factors in Computing Systems. Montreal, QC (2018). p. 1–12. 10.1145/3173574.3174039

[58] Weiss RS Park S. Recent updates on myopia control: preventing progression 1 diopter at a time. Curr Opin Ophthalmol. (2019) 30:215–9. 10.1097/ICU.0000000000000571 31033732

[59] Williams AM Muir KW Rosdahl JA. Readability of patient education materials in ophthalmology: a single-institution study and systematic review. BMC Ophthalmol. (2016) 16:133. 10.1186/s12886-016-0315-0 27487960 PMC4973096

[60] Ates HC Fiannaca A Folmer E. Immersive simulation of visual impairments using a wearable see-through display. In: The 9th International Conference on Tangible, Embedded, and Embodied Interaction. Stanford, CA (2015). p. 225–8. 10.1145/2677199.2680551

[61] Jones PR Ometto G. Degraded reality: using VR/AR to simulate visual impairments. In: 2018 IEEEWorkshop on Augmented and Virtual Realities for Good (VAR4Good). Reutlingen (2018). p. 1–4. 10.1109/VAR4GOOD.2018.8576885

[62] Jones PR Somoskeöy T Chow-Wing-Bom H Crabb DP. Seeing other perspectives: evaluating the use of virtual and augmented reality to simulate visual impairments (OpenVisSim). npj Digit Med. (2020) 3:32. 10.1038/s41746-020-0242-6 32195367 PMC7064490

[63] Krösl K Elvezio C Luidolt LR Hürbe M Karst S Feiner S. CatARact: simulating cataracts in augmented reality. In: 2020 IEEE International Symposium on Mixed and Augmented Reality (ISMAR). Porto de Galinhas (2020). p. 682–93. 10.1109/ISMAR50242.2020.00098

[64] Berger JW Madjarov B. Augmented reality fundus biomicroscopy: a working clinical prototype. Archiv Ophthalmol. (2001) 119:1815–8. 10.1001/archopht.119.12.1815 11735793

[65] Devalla SK Liang Z Pham TH Boote C Strouthidis NG Thiery AH. Glaucoma management in the era of artificial intelligence. Br J Ophthalmol. (2020) 104:301–11. 10.1136/bjophthalmol-2019-315016 31640973

[66] Chen JJ. Optical coherence tomography and neuro-ophthalmology. J Neuro Ophthalmol. (2018) 38:e5–8. 10.1097/WNO.0000000000000505 28266953

[67] Roodaki H Filippatos K Eslami A Navab N. Introducing augmented reality to optical coherence tomography in ophthalmic microsurgery. In: 2015 IEEE Q18 International Symposium on Mixed and Augmented Reality. Fukuoka (2015). p. 1–6. 10.1109/ISMAR.2015.15

[68] Tang N Fan J Wang P Shi G. Microscope integrated optical coherence tomography system combined with augmented reality. Opt Express. (2021) 29:9407–18. 10.1364/OE.420375 33820369

[69] Pan J Liu W Ge P Li F Shi W Jia L. Real-time segmentation and tracking of excised corneal contour by deep neural networks for DALK surgical navigation. Comput Methods Programs Biomed. (2020) 197:105679. 10.1016/j.cmpb.2020.105679 32814253

[70] Ong CW Tan MCJ Lam M Koh VTC. Applications of extended reality in ophthalmology: systematic review. J Med Internet Res. (2021) 23:e24152. 10.2196/24152 34420929 PMC8414293

[71] Rosenfeld JV Wong YT. Neurobionics and the brain-computer interface: current applications and future horizons. Med J Aust. (2017) 206:363–8. 10.5694/mja16.01011 28446119

[72] Fanlo Zarazaga A Gutiérrez Vásquez J Pueyo Royo V. Review of the main colour vision clinical assessment tests. Arch Soc Esp Oftalmol. (2019) 94:25–32. 10.1016/j.oftal.2018.08.006 30361001

[73] Aydindogan G Kavakli K Sahin A Artal P Ürey H. Applications of augmented reality in ophthalmology [invited]. Biomed Opt Express. (2021) 12:511–38. 10.1364/boe.405026 33659087 PMC7899512

[74] Atchison DA. Optics of the human eye. In: Guenther BD, Steel DG, editors. Encyclopedia of Modern Optics. 2nd ed. Vol. 5. Amsterdam: Elsevier (2018). p. 43–63.

[75] Shorgin S Samouylov K Gudkova I Galinina O Andreev S. On the benefits of 5G wireless technology for future mobile cloud computing. In: 2014 International Science and Technology Conference (Modern Networking Technologies) (MoNeTeC). Moscow (2014). p. 1–4. 10.1109/MoNeTeC.2014.6995601

[76] De Pace F Manuri F Sanna A. Augmented reality in industry 4.0. Am J Comput Sci Inform Technol. (2018) 6:1–7. 10.21767/2349-3917.100017

[77] Salih AE Elsherif M Ali M Vahdati N Yetisen AK Butt H. Ophthalmic wearable devices for color blindness management. Adv Mater Technol. (2020) 5:1901134. 10.1002/admt.20190113425855820

[78] Zhang S Tan H Rui X Yu Y. Vanadium-based materials: next generation electrodes powering the battery revolution? Acc Chem Res. (2020) 53:1660–71. 10.1021/acs.accounts.0c00362 32709195

