# Supplementary material for: Augmented Reality in Ophthalmology: Applications and Challenges
Source: Front Med (Lausanne). 2021 Dec 10;8:733241. doi: 10.3389/fmed.2021.733241 (PMC8703032; doi:10.3389/fmed.2021.733241)
Supplement: Supplementary file 1 [file Data_Sheet_1.PDF]

Supplementary Material

Supplementary Figures and Tables

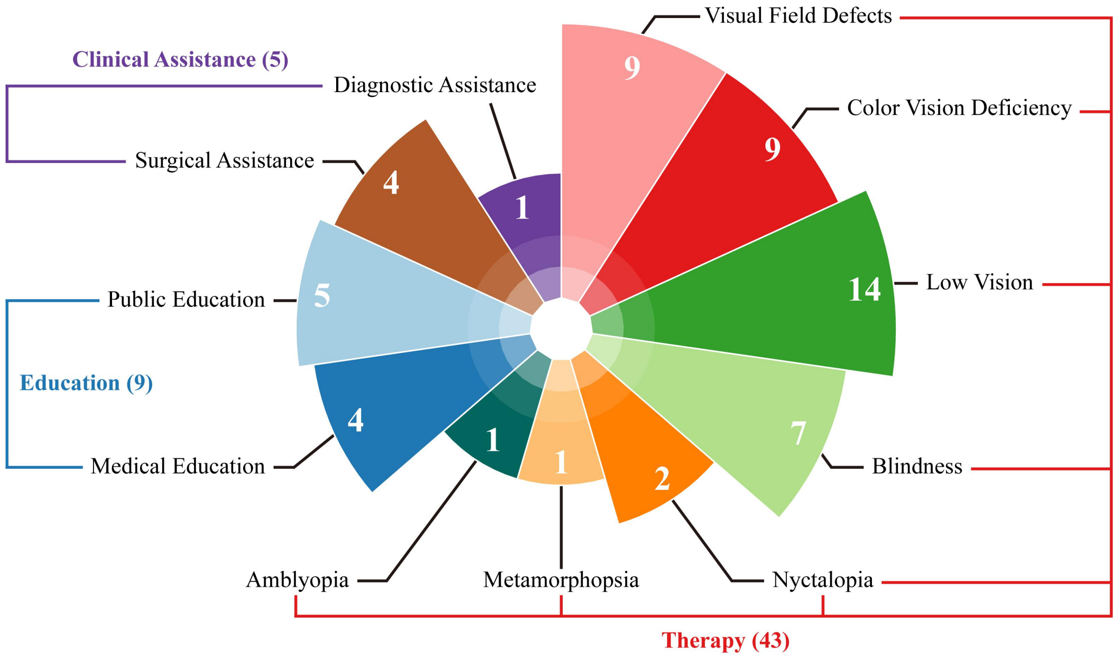

Supplementary Figure1. Publication statistics per application. The number of papers of every application overlay onto the sectors.

Supplementary Table1. Publications of AR's ophthalmic applications in Google Scholar database.

Primary classification	Secondary classification	Reference
Therapy (43)	Visual Field Defects (9)	Apfelbaum, H. L., Apfelbaum, D. H., Woods, R. L., and Peli, E. (2008). Inattentional blindness and augmented - vision displays: effects of cartoon - like filtering and attended scene. Ophthalmic and Physiological Optics, 28(3), 204-217. doi: 10.1111/j.1475-1313.2008.00537.x
		Ichinose, K., Fujishiro, I., Kashiwagi, K., Mao, X., Zhao, X., Toyoura, M., et al. (2020). Visual Field Loss Compensation for Homonymous Hemianopia Patients Using Edge Indicator. 2020 International Conference on Cyberworlds (CW), 79-85. doi: 10.1109/CW49994.2020.00019.
		Luo, G., and Peli, E. (2006). Use of an augmented-vision device for visual search by patients with tunnel vision. Investigative ophthalmology & visual science, 47(9), 4152-4159. doi: 10.1167/iovs.05-1672
		Sayed, A. M., Abdel-Mottaleb, M., Kashem, R., Roongpoovapatr, V., Elsawy, A., Abdel-Mottaleb, M., et al. (2020a). Expansion of Peripheral Visual Field with Novel Virtual Reality Digital Spectacles. American journal of ophthalmology, 210, 125-135. doi: 10.1016/j.ajo.2019.10.006
		Sayed, A. M., Kashem, R., Abdel-Mottaleb, M., Roongpoovapatr, V., Eleiwa, T. K., Abdel-Mottaleb, M., et al. (2020b). Toward Improving the Mobility of Patients with Peripheral Visual Field Defects with Novel Digital Spectacles. American journal of ophthalmology, 210, 136-145. doi: 10.1016/j.ajo.2019.10.005
		Vargas-Martin, F., & Peli, E. (2002). Augmented-view for restricted visual field: multiple device implementations. Optometry and Vision Science, 79(11), 715-723. doi: 10.1097/00006324-200211000-00009

		Younis, O., Al-Nuaimy, W., and Rowe, F. (2019). A hazard detection and tracking system for people with peripheral vision loss using smart glasses and augmented reality. International Journal of Advanced Computer Science and Applications, 10(2), 1-9. doi: 10.14569/IJACSA.2019.0100201
		Zhao, X., Go, K., Kashiwagi, K., Toyoura, M., Mao, X., and Fujishiro, I. (2019). Computational Alleviation of Homonymous Visual Field Defect with OST-HMD: The Effect of Size and Position of Overlaid Overview Window. 2019 International Conference on Cyberworlds (CW), 175-182. doi: 10.1109/CW.2019.00036.
		Zhao, Y., Szpiro, S., Knighten, J., and Azenkot, S. (2016). CueSee: exploring visual cues for people with low vision to facilitate a visual search task. 2016 ACM International Joint Conference on Pervasive and Ubiquitous Computing, 73-84. doi: 10.1145/2971648.2971730
	Color Vision Deficiency (9)	Dheeraj, K., Jilani, S. A. K., and JaveedHussain, M. S. (2015). Real-time Automated Guidance System to detect and Label Color for Color Blind People using Raspberry Pi. SSRG International Journal of Electronics and Communication Engineering, 2(11), 11-14. doi: 10.14445/23488549/IJECE-V2I11P103
		Fuller, T. L., & Sadovnik, A. (2017, September). Image level color classification for colorblind assistance. In 2017 IEEE International Conference on Image Processing (ICIP) (pp. 1985-1989). IEEE.
		Langlotz, T., Sutton, J., Zollmann, S., Itoh, Y., and Regenbrecht, H. (2018). Chromaglasses: Computational glasses for compensating colour blindness. Paper presented at the Proceedings of the 2018 CHI Conference on Human Factors in Computing Systems, 1-12. doi: 10.1145/3173574.3173964

		Lausegger, G., Spitzer, M., and Ebner, M. (2017). OmniColor--A Smart Glasses App to Support Colorblind People. International Journal Of Interactive Mobile Technologies, 11(5). doi: 10.3991/ijim.v11i5.6922
		Melillo, P., Riccio, D., Di Perna, L., Di Baja, G. S., De Nino, M., Rossi, S., et al. (2017). Wearable improved vision system for color vision deficiency correction. IEEE journal of translational engineering in health and medicine, 5, 1-7. doi: 10.1109/JTEHM.2017.2679746
		Popleteev, A., Louveton, N., and McCall, R. (2015). Colorizer: smart glasses aid for the colorblind. Proceedings of the 2015 workshop on Wearable Systems and Applications (pp. 7-8). doi: 10.1145/2753509.2753516
		Schmitt, S., Stein, S., Hampe, F., and Paulus, D. (2012). Mobile services supporting color vision deficiency. 2012 13th International Conference on Optimization of Electrical and Electronic Equipment (OPTIM), 1413-1420. doi: 10.1109/OPTIM.2012.6231860
		Tang, Y., Zhu, Z., Toyoura, M., Go, K., Kashiwagi, K., Fujishiro, I., et al. (2018). Arriving light control for color vision deficiency compensation using optical see-through head-mounted display. 16th ACM SIGGRAPH International Conference on Virtual-Reality Continuum and its Applications in Industry, 1-6. doi:10.1145/3284398.3284407
		Tanuwidjaja, E., Huynh, D., Koa, K., Nguyen, C., Shao, C., Torbett, P., et al. (2014). Chroma: a wearable augmented-reality solution for color blindness. 2014 ACM international joint conference on pervasive and ubiquitous computing, 799-810. doi: 10.1145/2632048.2632091
	Blindness (7)	Blum, J. R., Bouchard, M., and Cooperstock, J. R. (2011). What's around me? Spatialized audio augmented reality for blind users with a smartphone. The International Conference on Mobile and Ubiquitous Systems: Computing, Networking, and Services, 49-62. doi: 10.1007/978-3-642-30973-1_5

		Hicks, S. L., Wilson, I., Muhammed, L., Worsfold, J., Downes, S. M., and Kennard, C. (2013). A depth-based head-mounted visual display to aid navigation in partially sighted individuals. PloS one, 8(7), e67695. doi: 10.1371/journal.pone.0067695
		Joseph, S. L., Zhang, X., Dryanovski, I., Xiao, J., Yi, C., and Tian, Y. (2013). Semantic indoor navigation with a blind-user oriented augmented reality. 2013 IEEE International Conference on Systems, Man, and Cybernetics, 3585-3591. doi: 10.1109/SMC.2013.61
		Kinateder, M., Gualtieri, J., Dunn, M. J., Jarosz, W., Yang, X. D., and Cooper, E. A. (2018). Using an augmented reality device as a distance-based vision aid—promise and limitations. Optometry and Vision Science, 95(9), 727. doi: 10.1097/OPX.0000000000001232
		Liu, Y., Stiles, N. R., and Meister, M. (2018). Augmented reality powers a cognitive assistant for the blind. ELife, 7, e37841. doi: 10.7554/eLife.37841
		Mambu, J. Y. , Anderson, E. , Wahyudi, A. , Keyeh, G. , & Dajoh, B. . (2019). Blind Reader: An Object Identification Mobile- based Application for the Blind using Augmented Reality Detection. 2019 1st International Conference on Cybernetics and Intelligent System (ICORIS). doi: 10.1109/ICORIS.2019.8874906
		Sánchez, J., and Tadres, A. (2011). Augmented reality application for the navigation of people who are blind. International Journal on Disability and Human Development, 10(1), 75-79. doi: 10.1515/ijdhd.2011.015
	Low Vision (14)	Angelopoulos, A. N., Ameri, H., Mitra, D., and Humayun, M. (2019). Enhanced Depth Navigation Through Augmented Reality Depth Mapping in Patients with Low Vision. Scientific Reports, 9(1), 11230. doi: 10.1038/s41598-019-47397-w

		Bakshi, A. M., Simson, J., de Castro, C., Yu, C. C., an d Dias, A. (2019). Bright: an augmented reality assistive platform for visual impairment. The 2019 IEEE Games, Entertainment, Media Conference (GEM), 1-4. doi: 10.1109/GEM.2019.8811556
		Elgendy, M., Herperger, M., Guzsvinecz, T., and Lanyi, C. S. (2019). Indoor Navigation for People with Visual Impairment using Augmented Reality Markers. 2019 10th IEEE International Conference on Cognitive Infocommunications (CogInfoCom), 425-430. doi: 10.1109/CogInfoCom47531.2019.9089960.
		Gonçalves, P., Orlosky, J., and Machulla, T. K. (2020). An augmented reality assistant to support button selection for patients with age-related macular degeneration. 2020 IEEE Conference on Virtual Reality and 3D User Interfaces Abstracts and Workshops (VRW), 730-731. doi: 10.1109/VRW50115.2020.00216.
		Huang, J., Kinateder, M., Dunn, M. J., Jarosz, W., Yang, X. D., and Cooper, E. A. (2019). An augmented reality sign-reading assistant for users with reduced vision. PloS one, 14(1), e0210630. doi: 10.1371/journal.pone.0210630
		Hwang, A. D., and Peli, E. (2014). An augmented-reality edge enhancement application for Google Glass. Optometry and vision science: official publication of the American Academy of Optometry, 91(8), 1021. doi: 10.1097/OPX.0000000000000326
		Katz, B. F. G., Kammoun, S., Parseihian, G., Gutierrez, O., Brilhault, A., Auvray, M., et al. (2012). NAVIG: augmented reality guidance system for the visually impaired. Virtual Reality, 16(4), 253-269. doi: 10.1007/s10055-012-0213-6
		Lang, F., Schmidt, A., and Machulla, T. (2020). Augmented Reality for People with Low Vision: Symbolic and Alphanumeric Representation of Information. The International Conference on Computers Helping People with Special Needs, 146-156.doi: 10.1007/978-3-030-58796-3_19

		Min Htike, H., H. Margrain, T., Lai, Y. K., and Eslambolchilar, P. (2021). Augmented Reality Glasses as an Orientation and Mobility Aid for People with Low Vision: a Feasibility Study of Experiences and Requirements. 2021 CHI Conference on Human Factors in Computing Systems, 1-15. doi: 10.1145/3411764.3445327
		Moshtael, H., Tooth, C., Nuthmann, A., Underwood, I., and Dhillon, B. (2020). Dynamic text presentation on smart glasses: A pilot evaluation in age-related macular degeneration. British Journal of Visual Impairment, 38(1), 24-37. doi: 10.1177/0264619619889998
		Stearns, L., Findlater, L., and Froehlich, J. E. (2018). Design of an augmented reality magnification aid for low vision users. 20th International ACM SIGACCESS Conference on Computers and Accessibility, 28-39. doi: 10.1145/3234695.3236361
		Yoon, C., Louie, R., Ryan, J., Vu, M., Bang, H., Derksen, W., et al. (2019). Leveraging augmented reality to create apps for people with visual disabilities: A case study in indoor navigation. 21st International ACM SIGACCESS Conference on Computers and Accessibility, 210-221. doi:10.1145/3308561.3353788
		Zhao, Y., Szpiro, S., and Azenkot, S. (2015). Foresee: A customizable head-mounted vision enhancement system for people with low vision. 17th International ACM SIGACCESS Conference on Computers and Accessibility, 239-249. doi:10.1145/2700648.2809865
		Zhao, Y., Szpiro, S., Knighten, J., and Azenkot, S. (2016). CueSee: exploring visual cues for people with low vision to facilitate a visual search task. 2016 ACM International Joint Conference on Pervasive and Ubiquitous Computing, 73-84. doi: 10.1145/2971648.2971730

	Nyctalopia (2)	Fernandez, A., Fernandez, P., López, G., Calderón, M., and Guerrero, L. A. (2015). Troyoculus: An Augmented Reality System to Improve Reading Capabilities of Night-Blind People. The International Work-Conference on Ambient Assisted Living, 16-28. doi: 10.1007/978-3-319-26410-3_3
		Hu, C., Zhai, G., and Li, D. (2015). An Augmented-Reality night vision enhancement application for see-through glasses. 2015 IEEE International Conference on Multimedia and Expo Workshops (ICMEW), 1-6. doi: 10.1109/ICMEW.2015.7169860.
	Metamorphopsia (1)	Bozzelli, G., De Nino, M., Pero, C., and Ricciardi, S. (2020). AR Based User Adaptive Compensation of Metamorphopsia. Paper presented at the Proceedings of the International Conference on Advanced Visual Interfaces, 1-5. doi: 10.1145/3399715.3399929
	Amblyopia (1)	Nowak, A., Wozniak, M., Pieprzowski, M., and Romanowski, A. (2018). Towards amblyopia therapy using mixed reality technology. Paper presented at the 2018 Federated Conference on Computer Science and Information Systems (FedCSIS), 279-282. doi: 10.15439/2018F335
Education (9)	Medical Education (4)	Acosta, D., Gu, D., Uribe-Quevedo, A., Kanev, K., Jenkin, M., Kapralos, B., et al. (2018). Mobile e-training tools for augmented reality eye fundus examination. Interactive Mobile Communication, Technologies and Learning, 83-92. doi: 10.1007/978-3-030-11434-3_13
		Huang, Y. H., Chang, H. Y., Yang, W. L., Chiu, Y. K., Yu, T. C., Tsai, P. H., et al. (2018). CatAR: A Novel Stereoscopic Augmented Reality Cataract Surgery Training System with Dexterous Instruments Tracking Technology. 2018 CHI Conference on Human Factors in Computing Systems, 1-12. doi: 10.1145/3173574.3174039

		Ropelato, S., Menozzi, M., Michel, D., and Siegrist, M. (2020). Augmented reality microsurgery: a tool for training micromanipulations in ophthalmic surgery using augmented reality. Simulation in Healthcare, 15(2), 122-127. doi: 10.1097/SIH.0000000000000413
		Schuppe, O., Wagner, C., Koch, F., Manner, R. (2009). EYESi Ophthalmoscope–A Simulator for Indirect Ophthalmoscopic Examinations. Studies in health technology and informatics, 142, 295-300. doi: 10.3233/978-1-58603-964-6-295
	Public Education (5)	Ates, H. C., Fiannaca, A., and Folmer, E. (2015). Immersive simulation of visual impairments using a wearable see-through display. The ninth international conference on tangible, embedded, and embodied interaction, 225–228. doi: 10.1145/2677199.2680551
		Jakl, A., Lienhart, A. M., Baumann, C., Jalaeefar, A., Schlager, A., Schöffer, L., et al. (2020). Enlightening patients with augmented reality. 2020 IEEE Conference on Virtual Reality and 3D User Interfaces (VR), 195-203. doi: 10.1109/VR46266.2020.00038.
		Jones, P. R., and Ometto, G. (2018). Degraded reality: Using VR/AR to simulate visual impairments. 2018 IEEE Workshop on Augmented and Virtual Realities for Good (VAR4Good), 1-4. doi: 10.1109/VAR4GOOD.2018.8576885.
		Jones, P. R., Somoskeöy, T., Chow-Wing-Bom, H., and Crabb, D. P. (2020). Seeing other perspectives: evaluating the use of virtual and augmented reality to simulate visual impairments (OpenVisSim). npj Digital Medicine, 3(1), 32. doi: 10.1038/s41746-020-0242-6
		Krösl, K., Elvezio, C., Luidolt, L. R., Hürbe, M., Karst, S., Feiner, S., et al. (2020). CatARact: Simulating cataracts in augmented reality. 2020 IEEE International Symposium on Mixed and Augmented Reality (ISMAR), 682-693. doi: 10.1109/ISMAR50242.2020.00098.

Clinical Assistance (5)	Diagnostic Assistance (1)	Berger, J. W., and Madjarov, B. (2001). Augmented Reality Fundus Biomicroscopy: A Working Clinical Prototype. Archives of Ophthalmology, 119(12), 1815-1818. doi: 10.1001/archopht.119.12.1815
	Surgical Assistance (4)	Horvath, S. (2016). The optical coherence tomography microsurgical augmented reality system (OCT-MARS): A novel device for microsurgeries. [dissertation/docter's thesis]. [Pittsburgh (PA)]: Carnegie Mellon University
		Pan, J., Liu, W., Ge, P., Li, F., Shi, W., Jia, L., et al. (2020). Real-time segmentation and tracking of excised corneal contour by deep neural networks for DALK surgical navigation. Computer Methods and Programs in Biomedicine, 197, 105679. doi: 10.1016/j.cmpb.2020.105679
		Roodaki, H., Filippatos, K., Eslami, A., and Navab, N. (2015). Introducing augmented reality to optical coherence tomography in ophthalmic microsurgery. 2015 IEEE International Symposium on Mixed and Augmented Reality, 1-6. doi: 10.1109/ISMAR.2015.15
		Tang, N., Fan, J., Wang, P., and Shi, G. (2021). Microscope integrated optical coherence tomography system combined with augmented reality. Optics Express, 29(6), 9407-9418. doi:10.1364/OE.420375
